# Evaluation of the physical, chemical, antioxidant, and antibacterial properties of *Camellia oleifera* Abel. seed oil

**DOI:** 10.5114/bta/195496

**Published:** 2025-03-31

**Authors:** Pham My Hao, Luu Thao Nguyen, Tran Thi Mai Anh, Le Pham Tan Quoc

**Affiliations:** Institute of Biotechnology and Food Technology, Industrial University of Ho Chi Minh City, Ho Chi Minh City, Vietnam

**Keywords:** antibacterial activity, antioxidant activity, *Camellia oleifera*, fatty acids, oil

## Abstract

**Background:**

*Camellia* seeds are rich in oil and contain fatty acids that offer significant health benefits.

**Materials and methods:**

This study aimed to characterize *Camellia oleifera* seeds and their oil. Physical properties of the seeds, including 1000-seed weight, density, moisture content, specific gravity, and angle of repose, were determined. *Camellia* seed oil was extracted using the pressing method, and various physicochemical and biological properties of the oil—such as density, color, acid, peroxide, and saponification values, as well as antioxidant and antibacterial activities—were assessed.

**Results:**

Pressing the oil at 140^o^C yielded optimal results, achieving a recovery efficiency of 21.67%. Gas chromatography-mass spectrometry identified seven fatty acid components in the oil, with oleic acid (71.03%) being the most abundant. The antioxidant capacity of the oil was evaluated using a DPPH (2,2-diphenyl-1-picrylhydrazyl) assay, yielding an IC50 value of 265.8 mg/mL. However, the oil exhibited no inhibitory effects against four bacterial strains (*Pseudomonas aeruginosa, Escherichia coli, Bacillus cereus, and Staphylococcus aureus*).

**Conclusions:**

These findings highlight the potential of *Camellia* seed oil in food technology, particularly as an alternative to commercial cooking oils.

## Introduction

Oil is a vegetable fat commonly found in kitchens and widely used in food preparation, including frying, salads, doughs, and cakes (Calugar et al., 2024). It is consumed in large quantities annually, with popular varieties including palm oil, soybean oil, olive oil, peanut oil, and sesame oil. To diversify products, replace traditional oils, and provide proper nutrition, it is essential to explore cooking oil derived from various raw materials.

*Camellia oleifera* Abel. is a plant known for its oil, which holds high medicinal and nutritional value. It is primarily distributed in China, India, Japan, and Southeast Asian countries, thriving in subtropical regions. China accounts for over 90% of the global production of *C. oleifera* oil (Cheng et al., 2018; Zhu et al., 2019).

*Camellia* is a promising oil crop, offering a higher unsaturated fatty acid content (90%) than olive oil (86%) (Guo et al., 2018). Numerous studies have demonstrated its health benefits, including its role in preventing and treating cardiovascular and cerebrovascular diseases, reducing cholesterol levels, and protecting the liver (Bumrungpert et al., 2016; Yeh et al., 2020; Ko et al., 2019; Qiu et al., 2016). Additionally, *Camellia* seed oil (CSO) contains compounds such as sterols, squalene, tocopherol, and fatty acids that exhibit beneficial biological activities, including antiatherosclerotic properties and antioxidant capacity (Xiao et al., 2017; Zhang et al., 2019). CSO has been used as a functional food and dietary supplement to reduce cancer risk, support the digestive system, regulate blood cholesterol levels, and enhance immune function (Du et al., 2020; Wang et al., 2019). Furthermore, crude and refined CSO have shown antimicrobial activity against pathogens such as *Escherichia coli*, *Bacillus subtilis*, *Saccharomyces cerevisiae*, and *Aspergillus niger* (Zhou et al., 2014). These outstanding properties position CSO as a potential alternative to traditional cooking oils in food technology.

Despite its benefits, CSO is not yet widely popular in Asian countries, including Vietnam. However, *C. oleifera* represents a valuable raw material with high applicability. Every part of the plant, from the shell to the seeds and seed residue, can be utilized to produce various products for the medical, food, and industrial sectors (Zhang et al., 2011).

*Camellia* seeds grown in Lang Son province may yield results that differ from previous studies due to variations in tree varieties, soil conditions, and oil extraction methods. This study aims to evaluate the physical properties of *C. oleifera* seeds and the physicochemical and antioxidant properties of their oil. These criteria are crucial for assessing the quality of seeds and oils. The findings can provide valuable insights into the properties of oil produced in different regions and serve as a reference for consumers selecting edible vegetable oils.

## Materials and methods

### Materials, bacteria strains, and chemicals

*Camellia oleifera* seeds were harvested from Lang Son province, Vietnam (21°55’14.0”N, 106°48’40.0”E), in October 2023. *Camellia oleifera* trees typically reach harvest maturity 5–6 years after planting. The oil was extracted at 140, 150, and 160^o^C using an oil press machine (Yamafuji SY-168G, China). The extracted oil was filtered using cloth, bottled, and stored at room temperature.

The bacterial strains used in this study included *Pseudomonas aeruginosa*, *E. coli*, *B. cereus*, and *Staphylococcus aureus*. These strains were provided by the Institute of Biotechnology and Food Technology, Ho Chi Minh City University of Industry (Vietnam).

The chemicals used in this study included 2,2-diphenyl-1-picrylhydrazyl (DPPH; Sigma, USA) and dimethyl sulfoxide (DMSO; China). Culture and antibacterial media comprised Mueller Hinton Agar, Nutrient Broth (HiMedia, India), and other chemicals meeting analyticalgrade standards.

### Determination of the physical properties of Camellia seeds

The physical properties of *Camellia* seeds, including moisture content, 1000-seed weight, angle of repose, and bulk density, were determined following ISO 712 (1998) and Quoc (2021).

### Determination of the physical and physicochemical properties of Camellia seed oil

The color of the oil was measured directly using a colorimeter (model CR-400, Konika Minolta, Osaka, Japan), with the CIE color space model applied to interpret the following parameters: L^*^, lightness; a^*^, green/red value; and b^*^, blue/yellow value. The recovery yield was calculated as the percentage of oil weight relative to seed weight (% v/w). The density of the oil was measured according to ISO 6883 (2017b). Acid value, saponification value, and peroxide value were determined according to ISO 660 (2020), ISO 3657 (2023), and ISO 3960 (2017a), respectively.

### Gas chromatography-mass spectrometry (GC-MS) analysis of fatty acids of Camellia seed oil

Fatty acids in CSO were identified using the method outlined by Quyen and Quoc (2023). Samples were methylated using a rapid method involving ester conversion of neutral lipids through alkaline catalysis in anhydrous methanol (methyl conversion) with KOH as the reagent. Fatty acid compositions were analyzed using an Agilent Technology 5977E MSD with an autosampler and the Agilent 7820A GC system (Santa Clara, CA, USA). Chromatographic separation was performed on a Carbowax 20MTM column (30 m × 0.25 mm × 0.25 μm). A 0.2 μl sample was filtered, and a 0.1 μl sample was injected into a capillary gas chromatograph equipped with a split/nonlinear injection and a fused silica capillary column coated with various stationary phases. The temperature was maintained at 165^o^C for 3 min, ramped at a rate of 4^o^C/min to 195^o^C, and held at 195^o^C for 23 min. The total analysis time was 30 min. Fatty acids were identified by comparing the retention times of a standard mixture to those of the sample fatty acids and by matching them with entries in the NIST spectral library.

### Determination of the antioxidant capacity of Camellia seed oil

The antioxidant capacity (AC) of CSO was measured based on the method described by Quyen and Quoc (2023), with minor modifications. The oil was dissolved in ethanol (96%, v/v) to prepare solutions at varying concentrations. A 0.5 ml aliquot of the prepared solution was then mixed with 3.5 ml of 0.1 mM DPPH solution in ethanol. The mixture was kept in the dark for 30 min at 25^o^C.

The AC was determined by measuring the decrease in absorbance at 517 nm compared to a control sample. Butylated hydroxytoluene (BHT) served as the control. The percentage inhibition was plotted against the concentrations of the oil to determine the concentration required to achieve 50% inhibition (IC_50_). AC was calculated using the following formula:


$$\text{Inhibition}\;[\text{\%}]\;=\{\frac{{\text{A}}_{\text{DPPH}}–{\text{A}}_{\text{sample}}}{{\text{A}}_{\text{DPPH}}}\}\times 100$$ (1)

where *A_DPPH_* is the absorbance of a solution containing only DPPH solution, and *A_sample_* is the absorbance of the sample in the presence of DPPH.

### Determination of antibacterial activity of Camellia seed oil

The antibacterial activity (AA) of CSO was evaluated using the disc diffusion method described by Quyen and Quoc (2023), with minor modifications. Briefly, 100 μl of bacterial suspension (adjusted to a 0.5 McFarland standard, approximately 1.5 × 10^8^ CFU/ml) was spread evenly onto Mueller-Hinton agar (MHA) plates using sterile swabs. Sterile paper discs (6 mm in diameter) impregnated with 10 μl of oil were placed on the MHA plates. Gentamicin solution (10 μg/disc) and dimethyl sulfoxide (DMSO, 5%) served as positive and negative controls, respectively. Plates were incubated at 37^o^C for 24 h, and AA was assessed based on the size of the inhibition zone around each disc.

### Statistical data analysis

All experiments were performed in triplicate, and the results are expressed as the mean ± standard deviation (SD). Statistical significance was determined using one-way analysis of variance (ANOVA), followed by Fisher’s least significant difference (LSD) posthoc test to compare means (*p* < 0.05). Data analysis was conducted using Statgraphics Centurion XV software (version 15.1.02, Statgraphics Technologies, Inc., USA).

## Results and discussion

### Physical properties of Camellia seeds

Moisture content is a critical parameter influencing grain quality during storage (Gierz et al., 2022). As shown in Table 1, the moisture content of CS was relatively low at 6.6%, making them highly suitable for long-term internal storage. Similarly, Zhu et al. (2019) reported that storing CS at approximately 7% moisture content resulted in optimal quality. When compared to cereal grains such as corn (5.14%) and rice (5.3%) (Aremu et al., 2014), the moisture content of CS was slightly higher but not significantly so.

**Table 1:** Physical characteristics of *Camellia* seeds*

Properties	Values
Moisture [%]	6.6 ± 0.9
1000-seeds weight [g/1000 seeds]	527.6 ± 10.5
Bulk density [kg/m^3^]	559 ± 23
The angle of repose [°]	34.13 ± 0.59

* Camellia seeds were dehusked.

The 1000-seed weight in this study was 527.6 g, a value that can vary depending on moisture levels (Gierz et al., 2022). Compared to other oilseeds, such as sesame seeds (4.33 g) (Deivasigamani and Swaminathan, 2018) and guna seeds (41 g) (Aviara et al., 1999), *Camellia* seeds exhibited a much higher 1000-seed weight. This parameter is an indicator of grain quality, with larger values typically reflecting greater firmness and integrity of the seeds (Aremu et al., 2014).

The bulk density of CS was 559 kg/m^3^, a value that is significantly influenced by humidity. Bulk density is critical for determining seed volume during storage and serves as the basis for calculating the capacity of drying chambers, warehouses, and storage facilities. Another important factor in seed preservation is the angle of repose, which facilitates the design of agricultural machinery and equipment that relies on free-flowing materials, such as hoppers, loading buckets, and sliding pipes (Aremu et al., 2014). The angle of repose for CS was 34.13°, a relatively low value that simplifies the processes of design, storage, and transportation.

Currently, there is no published research on the physical properties of *C. oleifera* seeds, making direct comparisons with seeds from other regions is impossible. However, these findings provide valuable baseline data for future research.

### Physicochemical properties of Camellia seed oil

CSO is a pale yellow liquid with a characteristic taste. The oil’s quality is influenced by factors such as plant variety, environment, growing conditions, collection and preservation procedures, and pressing methods, leading to variations across studies.

As shown in Table 2, the color parameters of the oil were affected by pressing temperature. When the temperature increased from 140 to 160^o^C, the L* value decreased from 34.97 to 30.29, and the b* value dropped from 31.93 to 23.05. Changes in the a* value were negligible. Higher pressing temperatures resulted in darker oil with a burnt odor.

**Table 2:** Physicochemical properties of *Camellia seed* oil

Properties	Temperature [^o^C]		
	140	150	160
L*	34.97 ± 0.02^b^	35.94 ± 0.09^c^	30.29 ± 0.05^a^
a*	21.38 ± 0.06^b^	22.58 ± 0.04^c^	21.01 ± 0.01^a^
b*	31.93 ± 0.28^b^	32.96 ± 0.16^c^	23.05 ± 0.08^a^
Density [g/ml]	0.85 ± 0.01^a^	0.84 ± 0.01^a^	0.84 ± 0.01^a^
Peroxide value [meq/kg]	0.17 ± 0.0^a^	0.24 ± 0.0^b^	0.36 ± 0.0^c^
Acid value [mg KOH/g]	5.00 ± 0.03^a^	5.91 ± 0.08^b^	6.20 ± 0.19^c^
Saponification value [mg KOH/g]	195.62 ± 0.68^a^	197.46 ± 2.12^a^	196.11 ± 1.55^a^
Recovery yield [%, v/w]	21.67 ± 2.08^a^	20.00 ± 1.01^a^	21.11 ± 1.45^a^
State	Light yellow, bright characteristic odor	Light yellow, dark characteristic odor	Dark yellow, opaque burning odor

Different superscript lowercase letters (a–c) in the same row indicate significant differences (p < 0.05).

The recovery yield of CSO was relatively stable across the three pressing temperatures, fluctuating between 20% and 22%. Oil yield significantly depends on the extraction method. For instance, solvent extraction of *C. sinensis* seeds yields 23.85%, whereas the cold pressing method on a hydraulic press yields only 15.76% (Thao et al., 2021). The density of CSO also remained consistent, ranging from 0.84 to 0.85 g/ml, regardless of pressing temperature. With increasing pressing temperature, the acid value rose from 5 to 6.2 mg KOH/g, and the peroxide value increased from 0.17 to 0.36 meq/kg, indicating the oil’s susceptibility to oxidation at higher temperatures. The acid value of CSO exceeded that of peanut oil (4.40 mg KOH/g) and *C. sasanqua* oil (0.19–1.5 mg KOH/g) (Pandurangan et al., 2014; Thang et al., 2016). Additionally, the saponification value was relatively high, ranging from 195.62 to 197.46 mg KOH/g, suggesting potential for use in soap and shampoo production (Thao et al., 2021).

Based on these results, CSO extracted at 140^o^C is recommended for further experimentation. At this temperature, the oil was clear, bright yellow, and retained its characteristic odor. It exhibited the lowest acid and peroxide values while maintaining a stable recovery yield, highlighting its potential applications in food technology.

### Fatty acid composition of Camellia seed oil

The fatty acid composition of oil is a critical determinant of its classification and quality. As shown in Table 3, CSO contains seven fatty acids, with oleic acid (71.03%) as the most abundant, followed by palmitic acid (16.59%), stearic acid (5.38%), and linoleic acid (5.07%). These findings align with Chaikul et al. (2017), who reported that oleic acid accounted for the highest proportion of CSO (87.93%), though linoleic acid was minimal (0.1%). Differences in the fatty acid composition of CSO collected from various regions can be attributed to geographical, soil, and climatic variations. Additionally, variations in oil extraction methods contribute to differences in fatty acid content.

**Table 3:** Fatty acid composition of *Camellia seed* oil

No.	Fatty acid	Content [%]	RT. [min]
1	Palmitic acid	16.59	40.620
2	Linoleic acid	5.07	45.859
3	Oleic acid	71.03	46.069
4	Vaccinic acid	0.93	46.228
5	Stearic acid	5.38	46.901
6	Cis-11-Eicosenoic acid	0.33	52.029
7	Oleamide acid	0.68	57.667

Oleic acid is widely recognized for its health benefits. It supports numerous physiological functions and has demonstrated potential benefits for cancer, autoimmune and inflammatory diseases, infections, wound healing, and reducing cholesterol, total cholesterol, and blood sugar levels (Sales-Campos et al., 2013). According to Eckel et al. (2007), oils with high oleic acid content (50–65%) and linoleic acid content (20–30%) exhibit superior heat resistance during frying and minimize unpleasant flavors caused by oxidation or high temperatures.

Palmitic acid, which constitutes 16.59% of the oil, is the most prevalent saturated fatty acid in the human body. This composition underscores the significant potential of CSO for further exploration and utilization, particularly in the food industry, due to its health-promoting properties and functional benefits.

### Antioxidant capacity of Camellia seed oil

**Figure 1:**
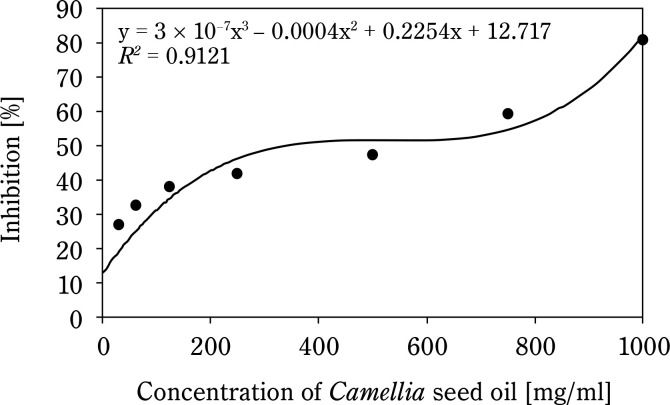
Antioxidant capacity of *Camellia seed* oil

**Figure 2:**
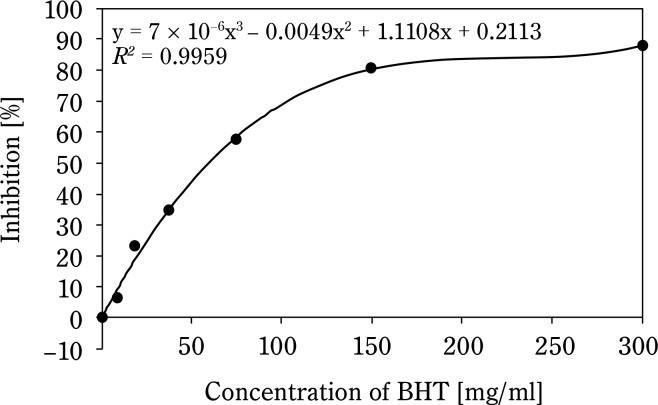
Antioxidant capacity of butylated hydroxytoluene (BHT)

Figure 1 shows that the AC of CSO increases with concentration. The IC_50_ of CSO, calculated from the regression equation, was 265.8 mg/ml, significantly higher than that of the BHT control sample (58.8 mg/ml) (Fig. 2). Compared to other vegetable oils, such as sesame oil (26 μg/ml) and sunflower oil (16.9 μg/ml) (Siger et al., 2008; Bopitiya and Madhujith, 2013), the IC_50_ of CSO was markedly higher, indicating a weaker AC. Furthermore, CSO from this study had a significantly lower AC compared to CSO from Ya’an City, China (IC_50_: 53.48 mg/ml) (Zhou et al., 2024). These findings suggest that the AC of oil depends on its chemical composition and extraction methods. According to Wu et al. (2008), high-pressing temperatures damage oil quality because heat can be a radical initiator, promoting the production of free radicals, speeding up the oxidation reaction, and decomposing or polymerizing the hydroperoxide. This underscores the limitations of the hot pressing method for oil extraction.

### Antibacterial ability of Camellia seed oil

Unlike some vegetable oils, such as sunflower oil (Alibe et al., 2012) and olive oil (Guo et al., 2020), CSO in this study exhibited no antibacterial ability (Table 4). This finding aligns with Wang et al. (2021), who reported that CSO from Hainan, China, had no inhibitory effect on *S. aureus*, *E. coli*, *P. aeruginosa*, *B. cereus*, or *B. subtilis*. However, their study also revealed that CSO from Guangxi, China, inhibited *E. coli* and *B. cereus* at high concentrations, even when extracted using the same physical pressing method.

**Table 4:** Antibacterial activity of *Camellia* seed oil

Microorganisms	Diameter of inhibition zone of gentamicin [mm]	Diameter of inhibition zone of *Camellia* seed oil [mm]
*Pseudomonas aeruginosa*	17.19 ± 0.86^a^	–
*Escherichia coli*	16.90 ± 0.49^a^	–
*Bacillus cereus*	21.94 ± 0.82^c^	–
*Staphylococcus aureus*	19.95 ± 0.28^b^	–

Different superscript lowercase letters (a–c) in the same column indicate significant differences (p < 0.05).

This variation highlights that AA is influenced by the chemical composition of the seeds, which is partly determined by plant origin and growing conditions. Additionally, the processing method plays a crucial role. Zhou et al. (2014) found that CSO extracted using cold pressing or organic solvent methods exhibited differing antibacterial properties.

Future research should explore these variations further, examining how oil extraction methods and storage conditions affect the biological activity of CSO.

## Conclusions

CSO was successfully extracted from *Camellia* seeds in Lang Son province, Vietnam. The evaluation of its physical, chemical, antioxidant, and antibacterial properties revealed unique physicochemical characteristics and chemical composition. The oil extracted at 140°C exhibited several optimal properties. Oleic acid, which accounted for the highest proportion, and linoleic acid, an essential fatty acid for human nutrition, were notable components of the CSO. The antioxidant capacity of CSO was determined, with an IC_50_ of 265.8 mg/ml. However, the oil showed no antibacterial activity against the four tested bacterial strains. Overall, CSO has strong potential as a vegetable-based cooking oil and could serve as an alternative to other vegetable oils due to its richness in fatty acids. The characteristics of CSO were influenced by various factors, particularly the extraction methods and the source of raw materials. These findings contribute valuable insights into the potential nutritional, cosmetic, and medical applications of CSO.
